# The Deep-Sea Microbial Community from the Amazonian Basin Associated with Oil Degradation

**DOI:** 10.3389/fmicb.2017.01019

**Published:** 2017-06-13

**Authors:** Mariana E. Campeão, Luciana Reis, Luciana Leomil, Louisi de Oliveira, Koko Otsuki, Piero Gardinali, Oliver Pelz, Rogerio Valle, Fabiano L. Thompson, Cristiane C. Thompson

**Affiliations:** ^1^Institute of Biology, Federal University of Rio de JaneiroRio de Janeiro, Brazil; ^2^Department of Chemistry, Florida International University, MiamiFL, United States; ^3^BP Exploration & Production Inc., HoustonTX, United States; ^4^SAGE/COPPE, Federal University of Rio de JaneiroRio de Janeiro, Brazil

**Keywords:** hydrocarbon, biodegradation, deep-sea, Amazon, bacteria, archaea, fungi

## Abstract

One consequence of oil production is the possibility of unplanned accidental oil spills; therefore, it is important to evaluate the potential of indigenous microorganisms (both prokaryotes and eukaryotes) from different oceanic basins to degrade oil. The aim of this study was to characterize the microbial response during the biodegradation process of Brazilian crude oil, both with and without the addition of the dispersant Corexit 9500, using deep-sea water samples from the Amazon equatorial margin basins, Foz do Amazonas and Barreirinhas, in the dark and at low temperatures (4°C). We collected deep-sea samples in the field (about 2570 m below the sea surface), transported the samples back to the laboratory under controlled environmental conditions (5°C in the dark) and subsequently performed two laboratory biodegradation experiments that used metagenomics supported by classical microbiological methods and chemical analysis to elucidate both taxonomic and functional microbial diversity. We also analyzed several physical–chemical and biological parameters related to oil biodegradation. The concomitant depletion of dissolved oxygen levels, oil droplet density characteristic to oil biodegradation, and BTEX concentration with an increase in microbial counts revealed that oil can be degraded by the autochthonous deep-sea microbial communities. Indigenous bacteria (e.g., *Alteromonadaceae, Colwelliaceae*, and *Alcanivoracaceae*), archaea (e.g., *Halobacteriaceae, Desulfurococcaceae*, and *Methanobacteriaceae*), and eukaryotic microbes (e.g., Microsporidia, Ascomycota, and Basidiomycota) from the Amazonian margin deep-sea water were involved in biodegradation of Brazilian crude oil within less than 48-days in both treatments, with and without dispersant, possibly transforming oil into microbial biomass that may fuel the marine food web.

## Introduction

Deepwater, offshore oil production may result in direct oil exposure to the marine environment by unplanned (oil spills) and planned release (as dispersed oil with operational discharge of produced water). Thus, it is important to evaluate the potential of indigenous microorganisms (both prokaryotes and eukaryotes) from different oceanic basins to degrade oil (Hazen et al., 2016). Microbes from all three domains of life can exploit certain compounds from the ∼600 1000 tons of oil that are annually released globally from (a) natural seeps on the seafloor, (b) delocalized runoff from human activities, and (c) oil spills (National Research Council (US) Committee on Oil in the Sea: Inputs, Fates, and Effects, 2003). In 2011, an oil well leak in the Campos Basin, 120-km off the coast of Rio de Janeiro, caused 3,700 barrels of oil to be released into the ocean (ANP, 2012). Recent studies have demonstrated the common presence of oil-degrading bacteria in several different deep-sea oil basins (Prince, 2005; Jurelevicius et al., 2013; Hazen et al., 2016). However, most studies have focused on assessing the bacterial community structure in the North Atlantic, Gulf of Mexico, and Mediterranean Sea. Because each oceanic basin has its own physicochemical and biological features, the geographic location may thus interfere in different ways with oil biodegradation rates. In addition, only a few studies have addressed the role of deep-sea eukaryotes (e.g., fungi) and archaea in biodegradation, as bacteria are considered the main player in oil biodegradation in the ocean (Hazen et al., 2016). The majority of studies addressing the role of fungi, protozoa, and archaea have focused on the terrestrial realm (McGenity et al., 2012).

Despite the ability of Halobacteriaceae to degrade oil compounds, including alkanes and polycyclic aromatic hydrocarbon (PAH), some archaeal groups appear to have reduced growth and diversity in the presence of oil (Röling et al., 2004). For example, *Nitrosopumilus maritimus* was sensitive to oil in experiments performed with seawater affected by the Deep Water Horizon (DWH) oil spill in the Gulf of Mexico in 2010 (Urakawa et al., 2012). However, Redmond and Valentine (2012) did not find a clear effect of oil on archaea growth and diversity (Redmond and Valentine, 2012), suggesting that further studies are needed to better understand the role of archaea in oil degradation (Al-Mailem et al., 2010; Tapilatu et al., 2010; Bonfá et al., 2011; Wang et al., 2011; Erdoǧmuş et al., 2013; Jurelevicius et al., 2014).

Fungi are known for their capacity to metabolize oil compounds, such as alkanes (Van Hamme et al., 2003), BTEX (Yadav and Reddy, 1993), and PAH (Cerniglia and Sutherland, 2001, 2006, 2010). The major phyla known to metabolize oil compounds are Microsporidia, Ascomycota, and Basidiomycota (Harms et al., 2011). Filamentous fungi make bacterial oil utilization by bacteria more efficient through the production of hyphae that act as continuous pathways for motile bacteria, linking oil aggregates (Kohlmeier et al., 2005; Wick et al., 2007; Banitz et al., 2011). Lignolytic and non-lignolytic fungi could metabolize PAH, but a few are able to grow with these compounds as the sole carbon and energy source (Cerniglia and Sutherland, 2010). The assemblage of fungi and bacteria appear to facilitate PAH biodegradation (Boonchan et al., 2000; Wang et al., 2012). However, a majority of studies on fungi that metabolize oil compounds were conducted in soils and sediments. The major phylum known to be a metabolizer of oil compounds, Basidiomycota, inhabits terrestrial environments and is rare in marine systems (Shearer et al., 2007). Little is known about fungi that can biodegrade oil in deep-sea water and the possible ecological relationships between fungi and bacterial communities (Leahy and Colwell, 1990; Harms et al., 2011; Passarini et al., 2011).

Environmental approaches designed to accelerate oil biodegradation are required to mitigate damage caused by oil spills. Dispersants are important oil spill-response tools for enhancing the biodegradation that are required to mitigate ecosystem impact caused by oil spills. They speed up the natural process of microbial oil degradation by dispersing oil into micron-sized droplets that become buoyant and dilute enough in the water column that the natural levels of biologically available nitrogen, phosphorus and oxygen are sufficient to start and sustain microbial growth. Dispersant application reduces the surface tension of the oil and creates droplets smaller than those formed by natural dispersions or releases from natural seeps, allowing for quicker biodegradation. The small size of the oil droplets formed also increases the surface area available for microbial colonization and significantly enhances biodegradation; this was one of the most important processes for the removal of the oil released from the DWH spill (Atlas and Hazen, 2011; Vilcáez et al., 2013; Meckenstock et al., 2014). The United State District Court for the Eastern District of Louisiana (2010) found that 3.19 million barrels of oil were released from the Macondo well in the Gulf of Mexico in 2010 (The United States District Court for the Eastern District of Louisiana, 2015). Approximately 1.8 million gallons of chemical dispersants, primarily Corexit 9500, were also injected at wellhead or applied to water surface. After a test period, dispersant was injected at the wellhead between May 15 and July 15 in an effort to prevent the formation of large surface slicks until the well could be capped (Operational Science Advisory Team, 2010). Shortly after two test periods, dispersant was injected continuously at the MC252 wellhead after May 15th, until the well was capped 61 days later on July 15th to retain the oil in the water column and prevent the formation of large surface slicks (Kujawinski et al., 2011). Recent laboratory studies have demonstrated that over a period of ∼60 days, the indigenous microorganisms in the Gulf of Mexico were able to completely degrade oil in presence of Corexit 9500 under controlled laboratory conditions (Wang et al., 2016). Corexit 9500 seems to accelerate the biodegradation process, increase the number of oil-degrading microorganisms, such as Oceanospirillales and Cycloclasticus, leading to the rapid degradation of hydrocarbons (Brakstad et al., 2015b). However, other recent studies indicated that dispersant may negatively affect oil-degrading bacteria at concentration of ∼19 μg/L of Corexit 9500 (Kleindienst et al., 2015). Thus, from previous studies it is not clear if Corexit9500 would and interfere in microbial community diversity and speed up oil biodegradation in the Equatorial margin.

The potential for biodegradation of oil by indigenous microorganisms (both prokaryotes and eukaryotes) has been evaluated in different oceanic basins but not in the Equatorial Atlantic Ocean (Hazen et al., 2016). Most oil production in Brazil (>2 Million barrels per day) occurs in deep-water oil fields, such as the Campus Basin (ANP, 2012). Oil companies are seeking new frontiers for oil exploration and production in Brazilian waters. Among these regions, the Brazilian equatorial margin, which spans from Rio Grande do Norte to the Amapa States, is currently being investigated through large environmental surveys that support the new oil exploration activities (Lourenço et al., 2016). Oil exploration in the Amazonian continental shelf began in the early 1960s, but oil production has been focused on the continent. An example is the Urucu Basin, which is known to produce high quality oil, mainly for the production of diesel and naphtha in Brazil. Gas is also a major product from the Amazon region (ANP, 2009). In the last few years, major exploration operations were initiated on the Amazonian continental shelf and basins by oil companies. In support of environmental risk assessments (fate of the oil) it is important to evaluate the potential of these deep-sea waters to biodegrade oil. The aim of this study was to characterize the microbial response (growth, composition, diversity) related to the process of oil degradation (with and without the dispersant Corexit9500), using deep-sea water from the Amazon equatorial margin. We performed two 48-day experiments and used metagenomics to uncover the microbial (taxonomic and functional) diversity. We also linked these molecular microbiology data and information to conventional analyses of various physical–chemical and biological parameters related to oil biodegradation.

## Materials and Methods

### Sampling Location

For the biodegradation experiments, seawater samples containing indigenous Brazilian off-shore microbial assemblages were collected under semi-sterile conditions from two geographical locations (Foz do Amazonas: E 587.672 and N 600.161; Barreirinhas: E 216.294 and N 9.800.847) according to methodologies described previously (Wang et al., 2016). From each location, two water samples were collected using a rosette as follows: (i) a deep-water sample below the chemocline, close to the seabed, at about 2570 m below the surface and (ii) a sample collected at about 10 m below the surface. Water from at about 10 m below the surface and a deep sea were immediately transferred to sterile containers and transported to the laboratory in the dark at 4°C.

### Experimental Design

Two identical experiments were performed using the deep-sea water from Foz do Amazonas and Barreirinhas. The experiments were performed at 4°C in the dark, lasted for 48 days and were monitored periodically at four time points (T0 = arrival in the laboratory; T1 = Day 0, the beginning of the experiment; T2 = Day 8; T3 = Day 24, and T4 = Day 48). Each experiment consisted of three treatments [(I) water-accommodated fraction (WAF), (II) chemically enhanced water-accommodated fraction (CEWAF), and (III) Control] prepared as described previously (Brakstad and Faksness, 2000). Briefly, the WAF treatment consisted of deep-sea water supplemented with Itaipu oil (at a loading rate of 0.1 mL/L). The relatively low oil rate was chosen to avoid total depletion of oxygen concentration in the experimental unities. Despite the numerous literature on anaerobic degradation of oil (e.g., Zengler et al., 1999, pp 266–269; Kniemeyer et al., 2007, pp 898–901; Musat et al., 2009, pp 209–219), we sought to reproduce similar oxygen concentration normally observed in the ocean. The 10-L WAF preparations were placed in a refrigerated chamber kept at 4°C in the dark and gently mixed (no-vortex) during 96 h. After settling with no mixing for 4 h, the WAF samples were taken without disturbing the oil slick on top and transferred to 500 mL capped flasks. The CEWAF treatment was prepared in a similar fashion but the Itaipu oil was premixed with the Corexit9500 dispersant before it was added to the saltwater container (1 mL dispersant to 25 mL oil; DOR 1:25). The CEWAF treatment was also kept in the refrigerated chamber at 4°C in the dark and mixed with a magnetic stirring rod for 18 h, followed by 4 h settling (no stirring) before samples were taken. Controls consisted of seawater without the addition of (dispersed) oil. After settling, all samples (WAF, CEWAF, and Controls) were distributed in 500 mL capped flasks and incubated in the dark at 4–5°C for a period of up to 48 days. In addition, 1-L samples were collected for microbial community assessment. No additional nutrients were added to the seawater samples. Aliquots of WAF, CEWAF, and Control were plated on the BD Difco^TM^ Bushnell-Haas mineral medium supplemented with 1% Itaipu oil. Inoculated plates were incubated at 4–5°C for 7 days (Supplementary Figure 5). Physical–chemical and biological analyses were performed at four time points during the experiment, using sacrificial bottles for each analysis.

### Microbial and Oil Droplets Counts

Prokaryotic cytometry counts were performed in triplicate as described previously using the fluorescent nucleic acid stain 4′,6-diamidino-2-phenylindole (DAPI) (Gregoracci et al., 2012). Eukaryotic cells and oil droplets were counted using a Flowcam. Samples (18 mL) were fixed with 2 mL of fixer (20% buffered formaldehyde).

### Inorganic Nutrients, Dissolved Oxygen, and Hydrocarbon Concentration (BTEX, Alkanes, and PAH)

Nitrate and phosphate were measured as described previously (Gregoracci et al., 2012). Dissolved oxygen was measured using an electrochemical electrode. The analysis of volatile aromatic hydrocarbons [Benzene, Toluene, Ethylbenzenes, and Xylene (BTEX)], alkanes, and PAHs was conducted by liquid-liquid extraction of the water samples using methylene chloride following by quantitative determination by gas chromatography–mass spectrometry (GC–MS) as described previously (Wang et al., 2016).

### Metagenomic Analysis

Chemically enhanced water-accommodated fraction, WAF, and control samples (1-L) were filtered through a 0.2 μm Sterivex and extracted using the Nucleospin kit protocol. Illumina paired end sequencing was performed for all samples. The generated data were pre-processed with fastq-mcf and paired-ends were merged with PEAR (Zhang et al., 2014). Taxonomic analysis was carried out using Focus (Silva et al., 2014). The data were submitted to Mgrast (Meyer et al., 2008) to annotate sequences and a functional analysis (Supplementary Tables 1, 2).

## Results

### Environmental Physical–Chemical and Biological Analyses

**FIGURE 1:**
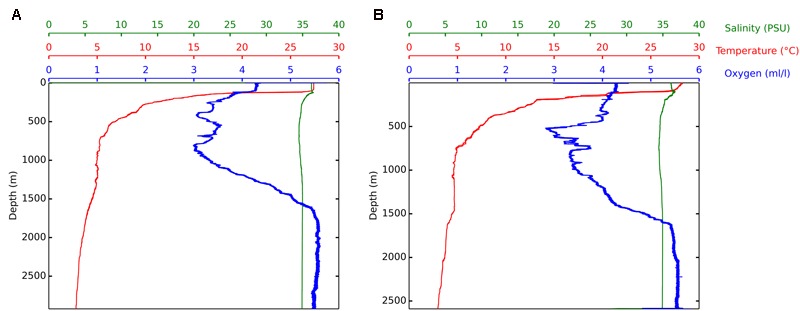
Features of the water masses. CTD measurements of temperature, salinity, and dissolved oxygen along depth. Water column profiles at Foz do Amazonas (E 587.672 and N 600.161) during the April, 2015 collection **(A)** and at Barreirinhas (E 216.294 and N 9.800.847) during the May, 2015 collection **(B)**.

Temperature, salinity, and oxygen profiles of both locations (Foz do Amazonas and Barreirinhas) had typical patterns with decreasing temperature and increasing oxygen concentration as the depth increased (**Figure 1**). The water samples used in this study correspond to the water mass named Deep Antarctic Waters (Wust, 1933) and had relative homogeneity with respect to temperature, salinity, and dissolved oxygen. The microbial community in the sea surface sample and deep-water sample at both locations was composed of different taxa related to oil degradation, including *Alteromonadaceae, Alcanivoracaceae*, and *Colwelliaceae* (Supplementary Figure 1).

### Biodegradation Experiments

Two experiments (Foz do Amazonas and Barreirinhas) were performed to evaluate the potential of deep-sea microbes to degrade crude oil during a 48-day incubation. The dynamics of physico-chemical and biological parameters were monitored by analyzing four time points (T0 = arrival; T1 = Day 0; T2 = Day 8; T3 = Day 24, and T4 = Day 48).

### Physico-Chemical Analysis

**FIGURE 2:**
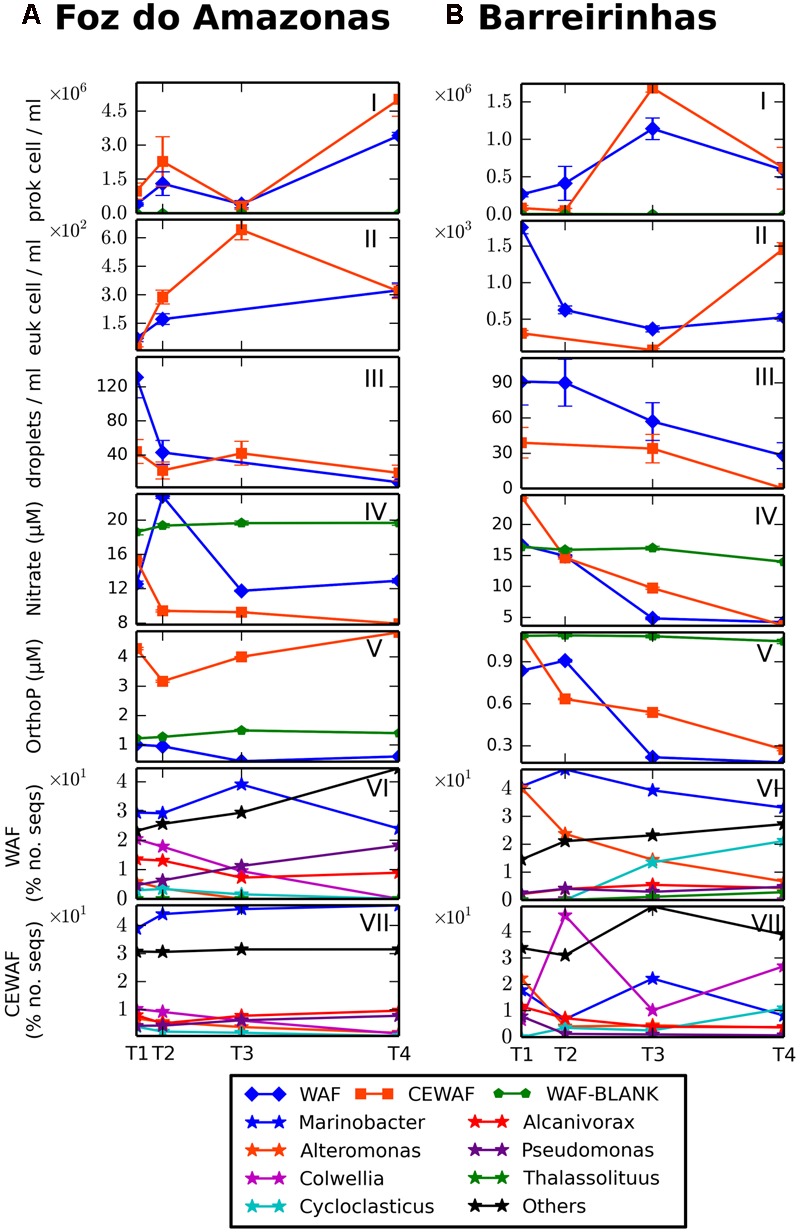
Microbial growth, oil droplets, nutrients and growth of major genera at Foz do Amazonas **(A)** and at Barreirinhas **(B)**. Prokaryotic cell counts (I), eukaryotic cell counts (II), oil dropplets counts (III), nitrate concentrations (IV), orthophosphate concentrations (V), metagenomic profiles of major genera in WAF treatment (VI), metagenomic profiles of major genera in CEWAF treatment (VII).

Oxygen concentration varied from ∼8 mg/l at T0 of the experiment to ∼6.2 and ∼6.6 mg/L at the end of the Foz and Barreirinhas experiments, respectively. In the Foz do Amazonas experiment, nitrate increased until day 8 (12.5 to 22.7 μM) and decreased to 12.9 μM after 48 days of the WAF treatment (**Figure 2A**). In the CEWAF treatment, nitrate dropped from 15.3 μM in the beginning of the experiment to 7.9 μM in the end. Orthophosphate varied from ∼1 μM (Day 0) to ∼0.6 μM (Day 48) and from 3.1 μM (Day 8) to 5 μM (Day 48) in the WAF and CEWAF treatments, respectively. In the Barreirinhas experiment, nitrate decreased from 16.7 to 4.2 μM and from 24.5 to 3.6 μM in the WAF and CEWAF treatments, respectively, toward the end of the experiment (**Figure 2B**). Orthophosphate had a similar trend, decreasing from 0.8 to ∼0.1 μM and from 1.1 to 0.2 μM in the end of the experiment, in the WAF and CEWAF treatments, respectively. The hydrocarbon concentration (BTEX, alkanes, and PAH) dropped significantly in both treatments, whereas the half-life of BTEX was 9 days for CEWAF treatment and 18 days for WAF treatment (Supplementary Table 3).

### Prokaryotic Counts

The abundance of prokaryotic cells in the Foz do Amazonas experiment varied from 3.85 × 10^5^ to 3.41 × 10^6^ cell/mL and from 9.72 × 10^5^ to 5.01 × 10^6^ cell/mL in WAF and CEWAF, respectively, with higher values occurring toward the end of the experiment (**Figure 2A**). In the Barreirinhas experiment, prokaryotic counts varied from 5.31 × 10^5^ to 2.28 × 10^6^ cell/mL and from 1.67 × 10^5^ to 3.36 × 10^6^ cell/mL in the WAF and CEWAF treatments, respectively, with the maximum count on day 24. Microbial counts were higher in the CEWAF than in the WAF treatment in both experiments.

### Eukaryotic Counts

In the Foz do Amazonas experiment, the total number of eukaryotes increased from 72 cell/mL and 17 cell/mL (Day 1) to 324 cell/mL (Day 48) and 642 cell/mL (Day 24) in the WAF and CEWAF treatments, respectively (**Figure 2A**). In the Barreirinhas experiments, the total number of eukaryotes dropped from 1754 cell/mL and 310 cell/mL (Day 1) to 369 cell/mL and 80 cell/mL (Day 24), in the WAF and CEWAF, respectively. An increase in eukaryotes number was observed in CEWAF treatment (1466 cell/mL) in Day 48.

### Oil Droplets

The number of oil droplets dropped throughout both experiments, reaching lower values in the CEWAF treatments (**Figures 2A,B**). In the Barreirinhas experiment, oil droplets dropped from 91 to 28 and from 39 to 0 droplets in WAF and CEWAF treatments, respectively.

### Taxonomic Assignment of Metagenomic Sequences

**FIGURE 3:**
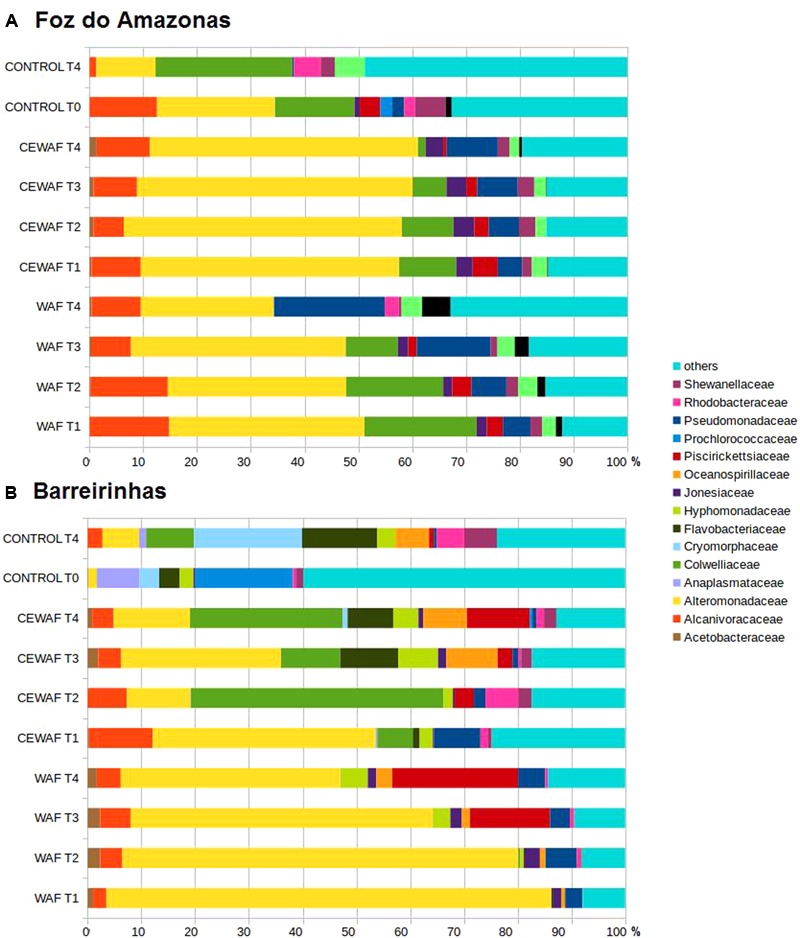
Bacterial community composition at family level for water treated with oil (WAF) and with dispersed oil (CEWAF) at days 8, 12, 24, and 48 determined by metagenomic analyses. Bacterial community for deep water control samples (no oil) are shown at days 0 and 48. Bacterial profiles at Foz do Amazonas **(A)** and at Barreirinhas **(B)**.

Overall, metagenomic sequences related to oil degrading bacteria, those are previous reported in the literature, corresponded to more than 60% of the total in WAF/CEWAF treatments of both experiments (**Figures 3A,B**). The major groups were *Alteromonadaceae* (*Alteromonas* e *Marinobacter*), *Colwelliaceae* (*Colwellia*), *Alcanivoracaceae* (*Alcanivorax*), *Piscirickettsiaceae* (*Cycloclasticus*), and *Oceanospirillaceae* (*Thalassolituus*) in both WAF and CEWAF. *Alcanivoracaceae* decreased from 14.51% (T1) to 8.93% (T4) and *Colwelliaceae* from 20.40% (T1) to 0% (T4) in the WAF treatment of Foz do Amazonas experiment. On the other hand, *Pseudomonadaceae* increased from 5.03% (T1) to 20.12% (T4). *Alteromonadaceae* peaked at T1 (35.50%) and T3 (39.08%). Other less abundant taxa, such as *Piscirickettsiaceae* (2.92 to 0%), *Shewanellaceae* (2.12 to 0.50%), and *Joneasiaceae* (1.86 to 0%) reached the lowest values at the end of the experiment, whereas *Ectothiorhodospiraceae* (1.13 to 5.17%) and *Vibrionaceae* (2.48 to 3.72%) tended to increase. Alteromonadaceae decreases from 80.98 to 39.3% in WAF treatment of Barreirinhas, whereas *Hyphomonadaceae* (0.64 to 4.84%), *Oceanospirillaceae* (0.68 to 2.88%), and *Piscirickettsiaceae* (14.23 to 22.74%) increased along time. *Alcanivoracaceae* and *Acetobacteraceae* peaked in T3 (5.41 and 2.22%, respectively); *Pseudomonadaceae* peaked at T2 (5.57%) and in T4 (4.87%). The control treatment (T0 and T4) in both experiments had a lower proportion of *Alteromonadaceae* and *Alcanivoracaceae* than in WAF/CEWAF treatments.

In the Foz do Amazonas experiment, CEWAF treatment had a greater proportion of *Marinobacter* than the WAF treatment (*p*-value < 0.01). *Marinobacter* increased from 38.89% (T1) to 47.17% (T4) in the CEWAF treatment; it peaked at T3 (39.08%) in the WAF treatment. *Colwellia* ranged from 10.32 to 1.42% in the CEWAF treatment and from 20.40 to 0% at T4 in the WAF treatment. *Pseudomonas* increased from 4.65% (T1) to 18.24% (T4) in WAF and from 4.26% (T1) to 7.83% (T4) in CEWAF. *Alcanivorax* was more abundant in the WAF treatment of the Foz do Amazonas experiment (*p*-value < 0.01).

The CEWAF treatment in the Barreirinhas experiment showed that *Alcanivoracaceae* (11.69 to 3.79%) and *Pseudomonadaceae* (8.47 to 0.68%) decreased over time whereas *Piscirickettsiaceae* increased from 0.007% (T1) to 11.07% (T4). *Colwelliaceae* increased at T2 (46.20%) and T4 (26.92%), *Alteromonadaceae* increased at T1 (40.96%) and T3 (27.46%), *Rhodobacteraceae* and *Shewanellaceae* increased at T2 (5.97 and 2.46%, respectively), and *Flavobacteriaceae, Hyphomonadaceae, Joneasiaceae*, and *Oceanospirillaceae* increased at T3 (9.93, 6.85, 1.42, and 8.80%, respectively). In the WAF, the most abundant group was *Alteromonadaceae* (∼80% at T1 and ∼45% at T4) and *Piscirickettsiaceae* (∼18% at T3 and ∼25% at T4).

Considering genera, in the CEWAF of Barreirinhas experiment, *Colwellia* ranged from 6.52% (T1) to 46.20% (T2). *Thalassolituus* peaked at T4 (7.02%). *Cycloclasticus* peaked at T4 (10.88%) in the CEWAF and at T3 (21.17%) in the WAF treatment. *Alcanivorax* decreased from 11.61% (T1) to 3.79% (T4) in CEWAF treatment whereas in the WAF it peaked at day 24 (5.41%) and dropped to 4.33% at day 48. The control treatment (T4) had a different microbial community profile in both experiments, with lower *Alteromonadaceae* and *Alcanivoracaceae* sequence abundance.

### Archaeal Groups

**FIGURE 4:**
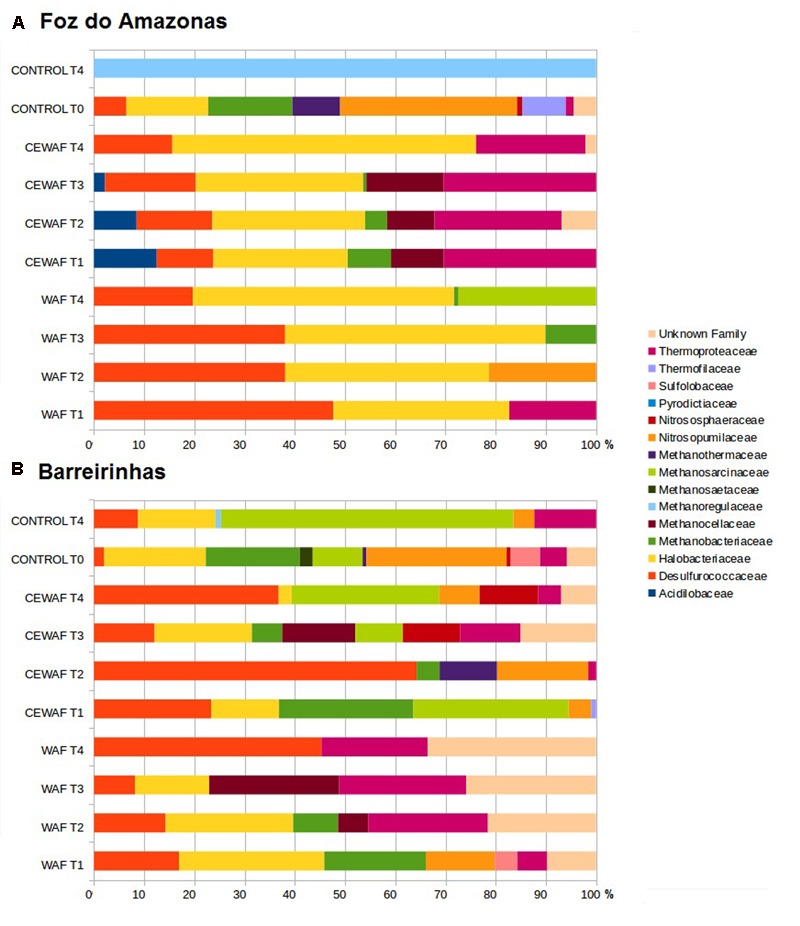
Archaeal community composition at family level for water treated with oil (WAF) and with dispersed oil (CEWAF) at days 8, 12, 24, and 48 determined by metagenomic analyses. Archaeal community for deep water control samples (no oil) are shown at days 0 and 48. Archaeal profiles at Foz do Amazonas **(A)** and at Barreirinhas **(B)**.

In the Foz do Amazonas experiment, the major groups were *Thermoproteaceae, Desulfococcus, Halobacteriaceae*, and *Methanobacteriaceae* in both WAF and CEWAF treatments (**Figure 4A**). The proportion of Desulfococcus decreased from 47.66% (T1) to 19.69% (T4) and proportion of Halobacteriaceae increased from 35.04% (T1) to 52.0% (T4) along time in the WAF treatment of the Foz experiment. *Thermoproteaceae* (17.29%), *Nitrosopumilaceae* (21.35%), *Methanobacteriaceae* (10.09%), and *Methanosarcinaceae* (27.40%) peaked in T1, T2, T3, and T4, respectively, in the WAF treatment. In CEWAF treatment, *Thermoproteaceae* (from 30.34 to 21.79%), *Acidilobaceae* (from 12.49 to 0%) and *Methanobacteriaceae* (from 8.62 to 0%) decreased, reaching the lowest values at T4 whereas *Halobacteriaceae* increased from 26.76% (T1) to 60.50% (T4) over time. In the control treatment, the major groups at T0 were *Nitrosopumilaceae* (35.21%), *Halobacteriaceae* (16.27%), and *Methanobacteriaceae* (16.75%), corresponding to ∼68% of total archaeal sequences, whereas the control treatment at T4 was dominantly *Methanoregulaceae*.

In the Barreirinhas experiment, the major groups were *Desulfococcus, Thermoproteaceae, Halobacteriaceae*, and *Nitrosopumilaceae*. *Desulforucoccaceae* increased in both WAF, from 16.94% (T1) to 45.28% (T4) and in CEWAF, from 23.30% (T1) to 64.25% (T2) (**Figure 4B**). *Sulfolobaceae* (4.51%) and *Nitrosopumilaceae* (13.72%) appeared only at T1 in WAF. Thermoproteaceae increased from 5.95% (T1) to 25.30% (T3) whereas Halobacteriaceae and Methanobacteriaceae decreased from 28.91% (T1) to 0% (T4) and from 20.21% (T1) to 0% (T3), respectively. In the CEWAF treatment, *Nitrosopumilaceae* peaked at T1 (13.72%) and T2 (18.15%), whereas *Methanobacteriaceae* decreased along time from 26.72% (T1) to 0% (T4). *Halobacteriaceae* (19.36%), *Thermoproteaceae* (11.98%) and *Methanocellaceae* (14.55%) peaked at T3. In the control treatment, *Nitrosopumilaceae* dropped from 27.93% (T0) to 4.17% (T4), Halobacteriaceae from 20.23% (T0) to 15.44% (T4) whereas *Methanosarcinaceae* (58.22%), *Desulfococcus* (8.78%), *Thermoproteaceae* (12.31%) and peaked at T4.

### Eukaryotes

**FIGURE 5:**
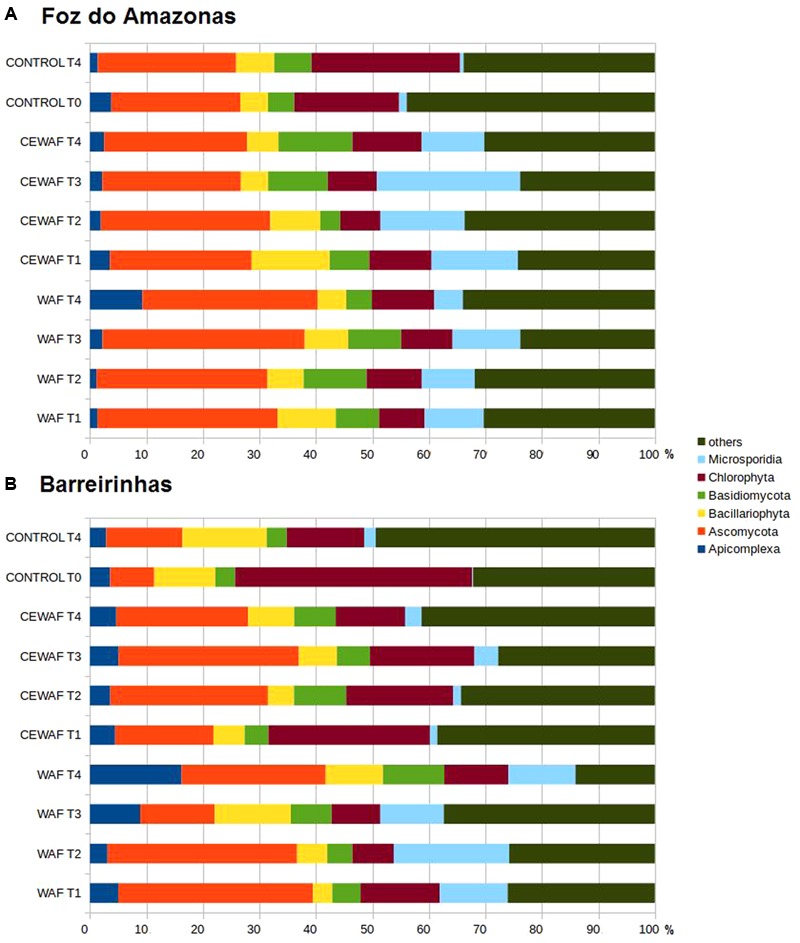
Eukaryotic community composition at family level for water treated with oil (WAF) and with dispersed oil (CEWAF) at days 8, 12, 24, and 48 determined by metagenomic analyses. Eukaryotic community for deep water control samples (no oil) are shown at days 0 and 48. Eukaryotic profiles at Foz do Amazonas **(A)** and at Barreirinhas **(B)**.

Apicomplexa, Ascomycota, Bacillariophyta, Basidiomycota, Chlorophyta, and Microsporidia were the major groups found (**Figures 5A,B**). There was a clear increase in Ascomycota (from 22 to 31% in the WAF treatment and 22 to 25% in the CEWAF treatment in seawater from Foz and from 7 to 34% in the WAF treatment and 7 to 17% in the CEWAF treatment in seawater from Barreirinhas) and Microsporidia (from 1 to 10% in the WAF treatment and 1 to 15% in the CEWAF treatment in seawater from Foz and from 0.1 to 12% in the WAF treatment and 0.1 to 1% in the CEWAF treatment in seawater from Barreirinhas) at T1 of both the WAF treatment and the CEWAF treatment in the two experiments compared to the T0. The same was observed for Basidiomycota (from 4 to 13% at T4 of CEWAF in the Foz do Amazonas experiment) and Apicomplexa (from 3 to 9% and from 3 to 16% in T4 in the WAF Foz and Barreirinhas experiments, respectively).

### Functional Identification of Metagenomics Sequences

**FIGURE 6:**
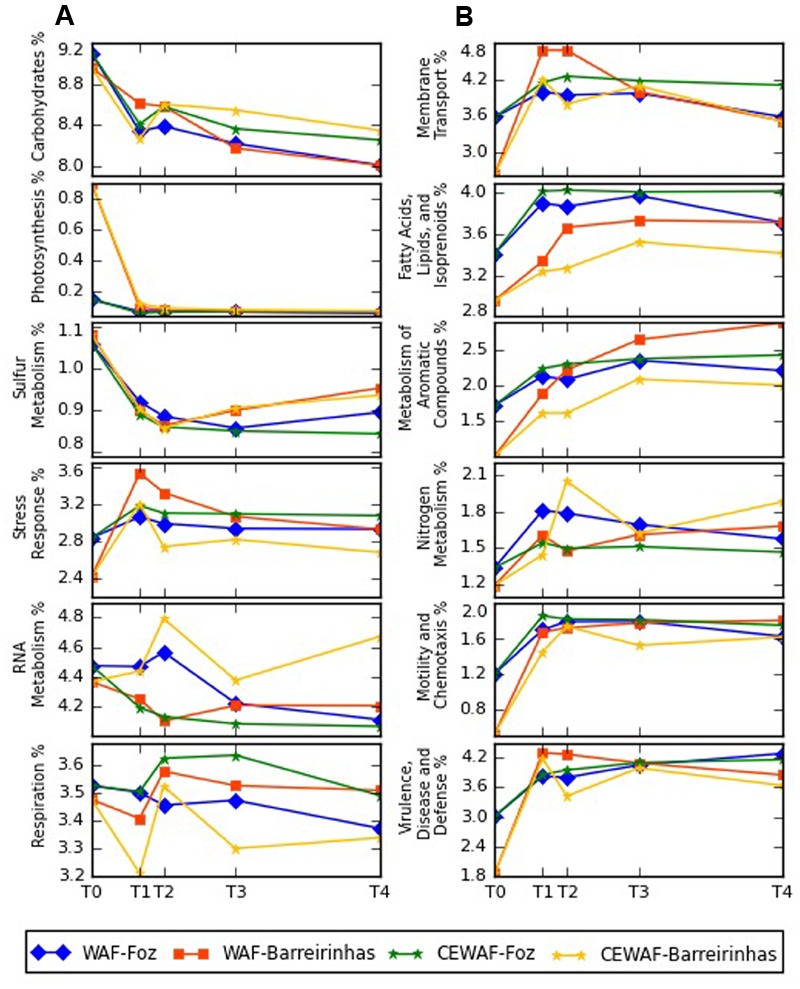
Major differences between subsystems profiles determined by metagenomic analyses at Foz do Amazonas **(A)** and at Barreirinhas **(B)**. T0 corresponds to control deep water samples (no oil) and T1, T2, T3, and T4 corresponds to days 8, 12, 24, and 48, respectively.

The functional profile of both treatments, WAF and CEWAF, was very similar (Supplementary Figures 2A,B). The major functional groups were carbohydrates, amino acids, proteins, cofactors and enzymes, representing over 60% of the metagenomic sequences with known function in both experiments. The number of sequences associated with membrane transport, metabolism of aromatics, motility and chemotaxis, virulence and disease increased over the time in both experiments while the number of sequences associated with carbohydrates and photosynthesis metabolism decreased (**Figure 6**).

The analysis related to genes for oil biodegradation showed that the abundance of sequences related to BTEX degradation increases along time in both experiments for both treatments, with more percentage of sequences in WAF treatment than in CEWAF and with a greater difference between WAF and CEWAF at Barreirinhas experiment. Alkanes proportion are constant along time in both treatments at Foz and have a peak at T2 and at T3, respectively, in WAF and CEWAF for Barreirinhas. PAH percentage of sequences in turn is greater for Barreirinhas, especially for WAF (Supplementary Figures 3A,B, 4A,B).

## Discussion

The physico-chemical parameters of deep-sea water from both locations examined were similar to the Antarctic deep-sea water masses (Silva et al., 2005). Oil biodegradation has been reported in different oceanic basins (e.g., Mediterranean Sea, Gulf of Mexico), under high oxygen concentrations and low temperatures (Hazen et al., 2010; Bargiela et al., 2015; Brakstad et al., 2015a,b), as observed in this study, indicating that oceanic conditions of these Brazilian deep-sea waters allow for oil degradation. The microbial communities disclosed in the sub-surface and deep-sea water of both locations analyzed here included several oil-degrading taxa (Yakimov et al., 2007), suggesting that indigenous microorganisms have the potential for biodegradation of oil. Despite slight differences in the taxonomic patterns between the Foz and Barreirinhas locations, major oil-biodegrading bacteria were found in all samples (e.g., *Alcanivoracaceae, Alteromonadaceae, Colwelliaceae, Pseudomonadaceae, Oceanospirillaceae*, and *Piscirickettisiaceae*).

### WAF and CEWAF Treatments Induce the Growth of Oil Degrading Microbes

The comparison of control with WAF/CEWAF treatments demonstrated the increase of microbes implicated with oil degradation throughout the 48-day experiments. For instance, during CEWAF treatment of Barreirinhas seawater, *Colwellia* boomed at day 8 (T2) and *Flavobacteriaceae* at day 24 (T3), whereas *Alcanivoracaceae* and *Alteromonadaceae* boomed at T1. Some groups peaked only after 24 days, for example, *Piscirickettsiaceae/Cycloclasticus*, and *Oceanospirillaceae/Thalassolituus*. During a 64-day biodegradation experiment with deep-sea water collected from the vicinity of the MC252 well in the Gulf of Mexico that was then amended with 10 μm oil droplets (2 ppm) created with Macondo Oil in the presence of Corexit 9500 dispersant, Wang et al. (2016) reported an increase in *Alteromonadaceae* (including *Marinobacter* species) and *Alcanivoracaceae* during the first 9 days. Furthermore, *Colwellia, Cycloclasticus*, and other Gammaproteobacteria increased between days 27 and 36, and Flavobacteria at day 64 (Wang et al., 2016). Hazen et al. (2010) found that Oceanospirillales dominated the oil plume samples from the DWH oil spill in GM and represented only a small fraction in the control sample out of the oil plume (Hazen et al., 2010). In our experiments, *Oceanospirillales* was not dominant. The differences between the studies may be related to the indigenous microbial communities of each location, differences in experimental set up, and the nature and composition of the oil used. Nevertheless, a clear positive response of oil on microbial biodegraders was observed in all studies.

In this study, the significant drop in oxygen concentration (from ∼8 mg/L at T0 to ∼6.2–6.6 mg/L at T4), possibly due to microbial respiration and degradation, with a concomitant increase in prokaryotic and eukaryotic counts, indicate the consumption of dispersed oil. *Colwellia* strains are known to degrade BTEX and alkane compounds (Methe et al., 2005) while *Alcanivorax* and *Marinobacter* are well-known alkane-degraders (Gauthier et al., 1992; Yakimov et al., 1998). During our experiments, *Marinobacter* abundance seemed to increase when *Colwellia* dropped and vice versa; both genera are able to degrade aliphatic hydrocarbons and could compete for similar resources. Alkane biodegradation related sequences proportions along time seems to show a trend similar to *Marinobacter* growth. Alkanes and BTEX are among the first oil compounds to be degraded (Hazen et al., 2010; Brakstad et al., 2015a; Wang et al., 2016); whereas PAH are the least easily degraded oil compound compared to alkanes and BTEX (Mason et al., 2014b). Cycloclasticus (*Piscirickettsiaceae*), an important PAH-degrading bacteria (Dyksterhouse et al., 1995), was mainly observed toward the end of our experiments. In turn, PAH biodegradation related sequences proportions along time is similar to growth pattern of Cycloclasticus. The capacity of microbial communities to metabolize hydrocarbons is also likely to be coupled with nitrogen availability (Mason et al., 2014b). A drop in nitrate concentration in both the Foz and Barreirinhas experiments suggest an intense denitrification process, possibly mediated by *Colwellia* (Methe et al., 2005; Mason et al., 2014a) and *Marinobacter* (Gauthier et al., 1992). In addition, a major component of the dispersant Corexit is dioctyl sodium sulfosuccinate (DOSS) (Place et al., 2010, 2014; Kujawinski et al., 2011). DOSS is a substrate that could be metabolized by *Colwellia* (Kleindienst et al., 2015).

### Oil-Degrading Archaea

Halobacteriaceae was one of most important groups in our experiment in line with previous studies (Tapilatu et al., 2010; Bonfá et al., 2011; Wang et al., 2011; Al-Mailem et al., 2012; Erdoǧmuş et al., 2013; Jurelevicius et al., 2014). However, this family was not the most abundant among the most important groups in both experiments. Some chemolithotrophic archaea that are able to oxidize organic sulfur compounds, such as *Desulfurococcaceae* and *Thermoproteaceae* (Burggraf et al., 1997; Zillig et al., 1982), were abundant, as well as methanogenic archaea, including Methanobacteriaceae, *Methanocellaceae, Methanoregulaceae, Methanosaetaceae, Methanosarcinaceae*, and *Methanotermaceae* (Garcia et al., 2000; Sakai et al., 2008, 2012). Methanogenic archaea were observed to co-occur with *Halobacteriaceae*, known as an oil degrading archaea, which is in accord with previous reports that methanogenesis is coupled to oil biodegradation in syntrophic communities (Zengler et al., 1999; Chang et al., 2006; Mayumi et al., 2016). Nevertheless, *Halobacteriaceae*, without methanogenic archaea only occurs with sulfur chemolithotrophic archaea in the CEWAF treatment (with dispersant). The presence of methanogenic archaea could imply that experiments reached anaerobic conditions. However, several methanogenic archaeal species (e.g., Halobacteriaceae) are aerobic and facultative anaerobic. The oxygen levels dropped to nearly 6 ml/L along both experiments (Supplementary Table 3). Although the oxygen levels were relatively high, it is possible that a suboxic microenvironment could be generated around the particles where these archaeal groups grows. This anaerobic microenvironment would be a result of intense respiration of the co-occurring groups observed with these archaea (oil degrading archaea or sulfur chemolithotrophic archaea). A remarkable difference between CEWAF treatments in the Foz and Barreirinhas experiments is the presence of *Nitrososphaera*, an ammonia oxidizing chemolithotrophic archaea (Könneke et al., 2005). The relationship between chemolithotrophy and oil degradation is unclear.

### Oil-Degrading Eukaryotes

Our experiments demonstrated that in both treatments, WAF and CEWAF, the growth of fungi (Microsporidia, Ascomycota, and Basidiomycota) was induced. Microsporidia, a PAH metabolizer, increased at T3, except for in the WAF treatment for Barreirinhas, where the PAH degrading bacteria, *Cycloclasticus*, peaked. Ascomycota is extremely versatile and metabolizes alkanes, BTEX, and PAHs (Harms et al., 2011). The abundance of this group was high and stable throughout the experiment, suggesting that several different oil compounds were used by Ascomycota. Basidiomycota abundance was lower than Microsporidia and Ascomycota abundance most of the time in both experiments. However, Basidiomycota abundance tended to increase toward the end of the experiment, having greater proportion than the Control. Although Basidiomycota can metabolize PAHs (Cerniglia and Sutherland, 2001, 2006, 2010; Sutherland, 2004; Verdin et al., 2004), this fungi generally cannot use PAHs as the sole carbon and energy source (Cerniglia and Sutherland, 2001). The absence of an additional nutrient source in our experiments may have influenced the utilization of PAH by Basidiomycota. Fungi produce multifunctional enzymes involved in the degradation of a large variety of organic molecules, including aromatic hydrocarbons, chlorinated organics, polychlorinated biphenyls, nitrogen-containing aromatics, and many other pesticides, dyes, and xenobiotics (Harms et al., 2011). Meanwhile, fungi and bacteria may establish partnerships to more efficiently degrade oil. Some positive correlations between the abundance of bacterial (e.g., *Alcanivoracaceae* and *Alteromonadaceae*) and fungi (e.g., *Ascomycota* and *Basidiomycota*) metagenomics sequences were observed in our study (Supplementary Figures 6, 7).

### WAF and CEWAF Treatments Influence the Microbial Metabolic Profiles

The availability of dispersed oil as the sole carbon source added for microbial growth from colonization of the buoyant oil droplets during the experiments resulted in changes in the functional profiles of both WAF/CEWAF treatments. The observed reduction in the carbohydrate metabolism and concomitant increase in metabolism of aromatic compounds, membrane transport, fatty acids, lipids and isoprenoids, motility and chemotaxis, nitrogen metabolism in WAF/CEWAF hints at important changes in the microbial communities that were driven by the presence of (dispersed) oil and dissolved oil constituents in our incubation experiments. One of the first steps in oil degradation is particle colonization and protein secretion on the particle surface mediated by trans-membrane proteins; furthermore, motility and chemotaxis allow prokaryotes to recognize and colonize oil droplets. We observed a drastic drop in oil droplets throughout the experiments. This decrease is likely mediated by colonization (Wang et al., 2016). The increase in aromatic compound metabolism highlights the biodegradation of different types of hydrocarbon molecules, including the oil components BTEX and PAH, whereas the increase in lipid and isoprenoid metabolism indicates an increased potential for alkane biodegradation. Hydrocarbonoclastic bacteria synthesize fatty acids, lipids and isoprenoids in the presence of *n*-alkanes (Kalscheuer et al., 2007). These lipids accumulate on the inside of the cells and are utilized as carbon source for growth, particularly in presence of a nitrogen source (Manilla-Pérez et al., 2010, 2011). However, we have no prove yet that the breakdown of alkanes is linked to an increase in lipid and isoprenoid biosynthesis.

The use of dispersants in oil spill accidents has been highly controversial (Kleindienst et al., 2015). In the present study, the GC–MS- results showed that the half-life of BTEX dropped from 18.5 to 9.4 days in the dispersant-oil treatment (CEWAF) at Barreirinhas experiment, suggesting a significant increase in the degradation of these aromatic compounds in the presence of Corexit9500. However, the bacterial community diversity results are not conclusive; while WAF T1, CEWAF T1 and Control T0 have very similar profiles in the Foz do Amazonas when compared to the Barreirinhas (**Figures 3A,B**). CEWAF had lower diversity compared to WAF in both experiments, suggesting that the dispersant may reduce microbial community diversity, and thus have a negative effect in the water quality. The dispersant appears to induce the growth of certain types of microbes (e.g., Colwelliaceae, Flavobacteriaceae, and Oceanospirillaceae), that increased at CEWAF in the Barreirinhas experiment compared to WAF. Furthermore, the BTEX, alkanes, and PAHs gene counts were higher in the WAF than in the CEWAF treatments, particularly in the Barreirinhas experiments (Supplementary Figures 3, 4), suggesting that the dispersant may actually inhibit the growth of certain important oil degrading microbes. This inhibition results on lower gene counts in the CEWAF treatments.

This study hints at possible roles for bacteria, archaea, fungi, and other microscopic eukaryotes in the Amazonas/Barreirinhas deep-sea water in oil biodegradation. We observed that chemolithotrophy, methanogenesis, nitrogen metabolism, and utilization of sulfur compounds are coupled to oil biodegradation (with and without addition of dispersants). Links between these processes need to be further elucidated, although some clues indicate their importance and coupling during oil degradation. It is also possible that the observations about the oil-degrading archaea, bacteria, and eukaryotes indicate an even larger network of an oil biodegradation community that also use metabolites from each other. Considering especially the multifunctional enzymes from fungi that partially degrade oil compounds, further efforts to known how microbial community structure itself are needed. This study demonstrates that the deep-sea water of the Amazonian margin contains indigenous prokaryotic and eukaryotic microbes that are able to degrade Brazilian crude oil within less than 48-days under controlled experimental conditions. The concomitant depletion of oil droplets and BTEX concentration with an increase in microbial counts suggests that oil can be transformed into microbial biomass and then enter the marine food chain. This study did not explicitly evaluate the toxicity of the dispersant Corexit 9500 on the microbial communities; however, from our data, it is possible to conclude that at least part of the dispersant may be used as a food resource by some deep-sea microbes from the Amazonian margin.

## Author Contributions

FT, MC, LR, PG, OP designed research; MC, LR, LL, LdO, KO, RV performed research; FT, MC, PG analyzed data; FT, MC, PG, OP, CT wrote the paper.

## Conflict of Interest Statement

The authors declare that the research was conducted in the absence of any commercial or financial relationships that could be construed as a potential conflict of interest.
