# Supplementary material for: The Deep-Sea Microbial Community from the Amazonian Basin Associated with Oil Degradation
Source: Front Microbiol. 2017 Jun 13;8:1019. doi: 10.3389/fmicb.2017.01019 (PMC5468453; doi:10.3389/fmicb.2017.01019)
Supplement: Supplementary file 1 [file Data_Sheet_1.doc]

Supplementary Material

THE DEEP-SEA MICROBIAL COMMUNITY FROM THE AMAZONIAN BASIN ASSOCIATED WITH OIL DEGRADATION

**Mariana E. Campeão^1+^, Luciana Reis^1+^, Luciana Leomil^1^, Louisi de Oliveira^1^, Koko Otsuki^1^, Piero Gardinali^2^, Oliver Pelz^3^, Rogerio Valle^2^, Fabiano L. Thompson^1,2*^, Cristiane C. Thompson^1*^**

^1^Institute of Biology and ^2^SAGE/COPPE. Federal University of Rio de Janeiro (UFRJ), Rio de Janeiro, Brazil. +, the two authors contributed equally for this work.

^2^Department of Chemistry. Florida International University (FIU), Miami, United States.

^3^BP Exploration & Production Inc., Houston, Texas, United States..

*** Correspondence:** Corresponding Author: fabianothompson1@gmail.com

**Supplementary Figure 1.** **Bacterial diversity.**
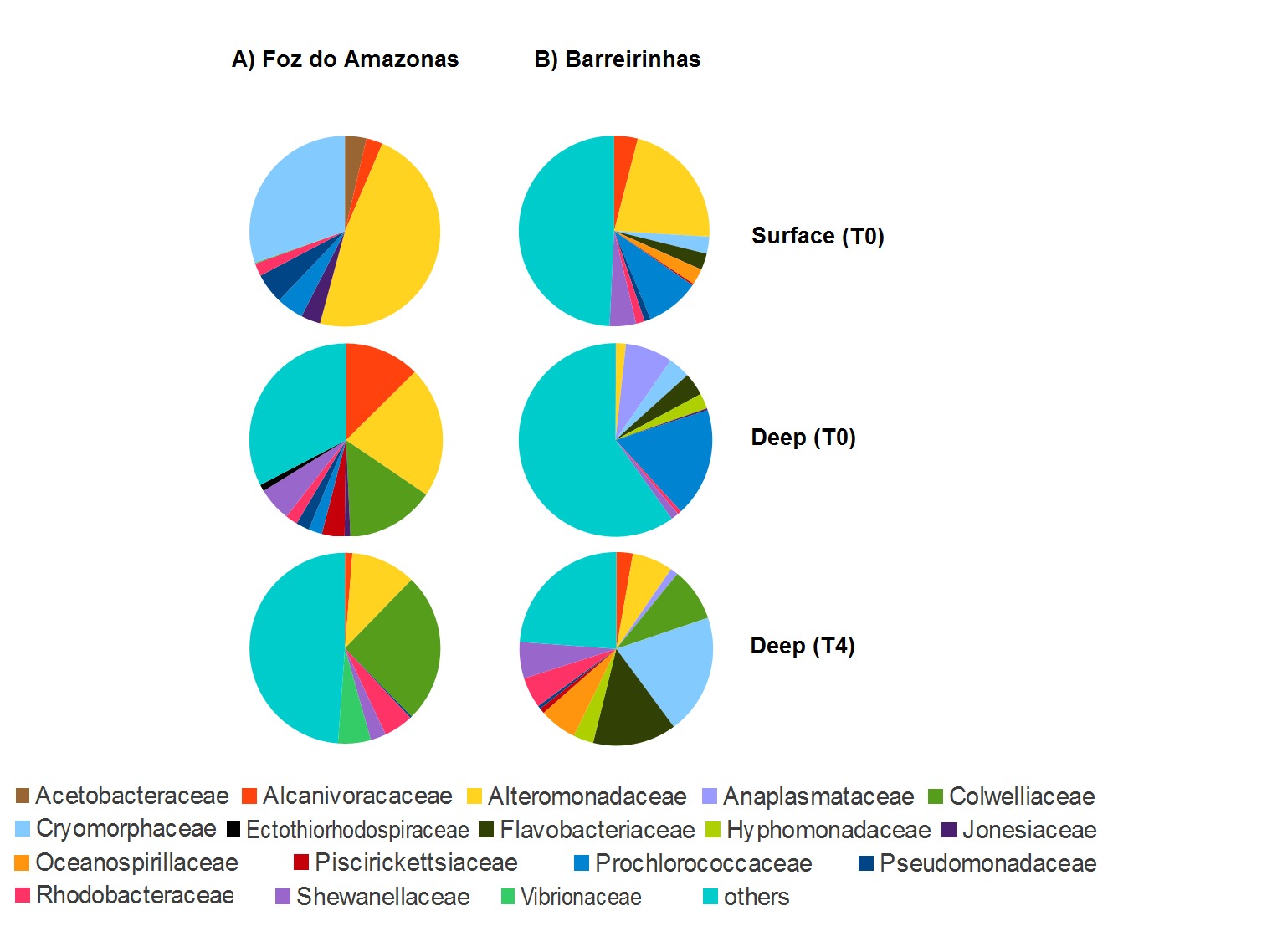
 Comparison of sub-surface and deep seawater bacterial community diversity from the two locations (Foz and Barreirinhas) and in different time points (T0=arrival in the laboratory; T4=end of experiments, day 48).

**Supplementary Figure 2.**
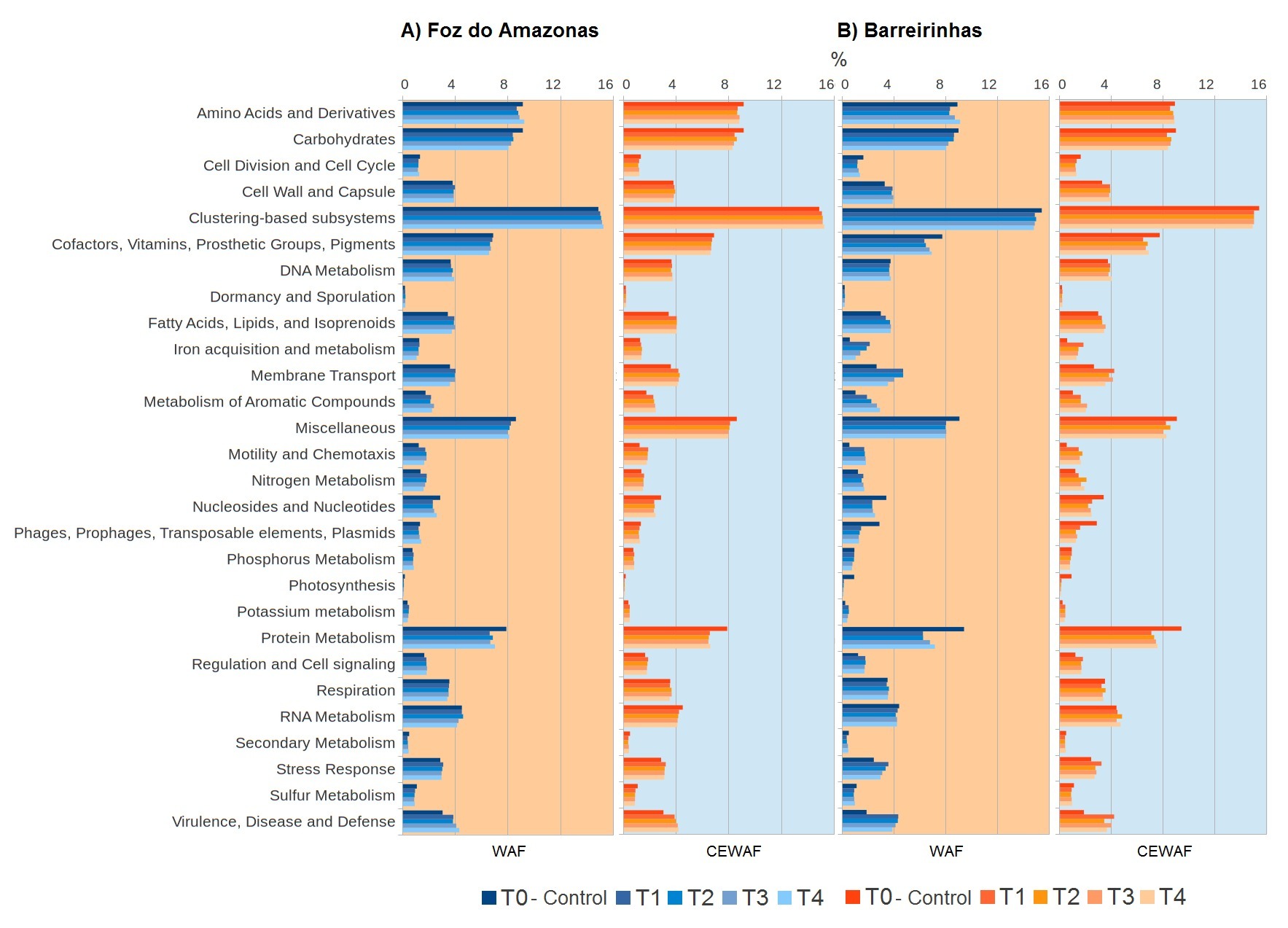
Subsistems profiles determined by metagenomic analyses at Foz do Amazonas (A) and at Barreirinhas (B). T0 corresponds to control deep water samples (no oil) and T1, T2, T3 and T4 corresponds to days 8, 12, 24 and 48, respectively. WAF treatment is represented in blue color bars and CEWAF in orange color bars.


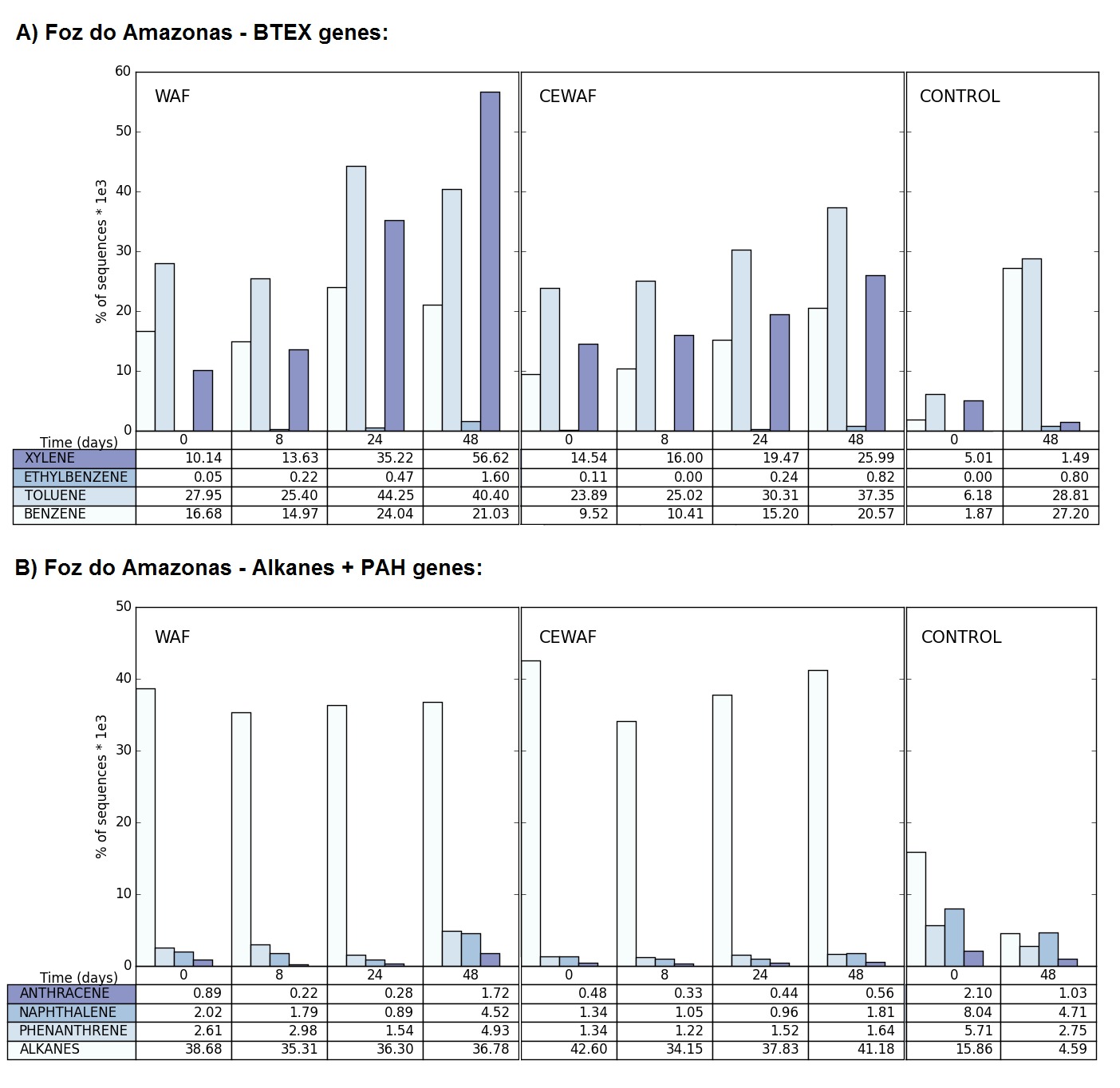


**Supplementary Figure 3.** Proportion (% of sequences X 10^3^) of oil biodegradation related genes determined by metagenomic analyses for BTEX degradation (A) and for alkanes and PAH degradation (B) at Foz do Amazonas.


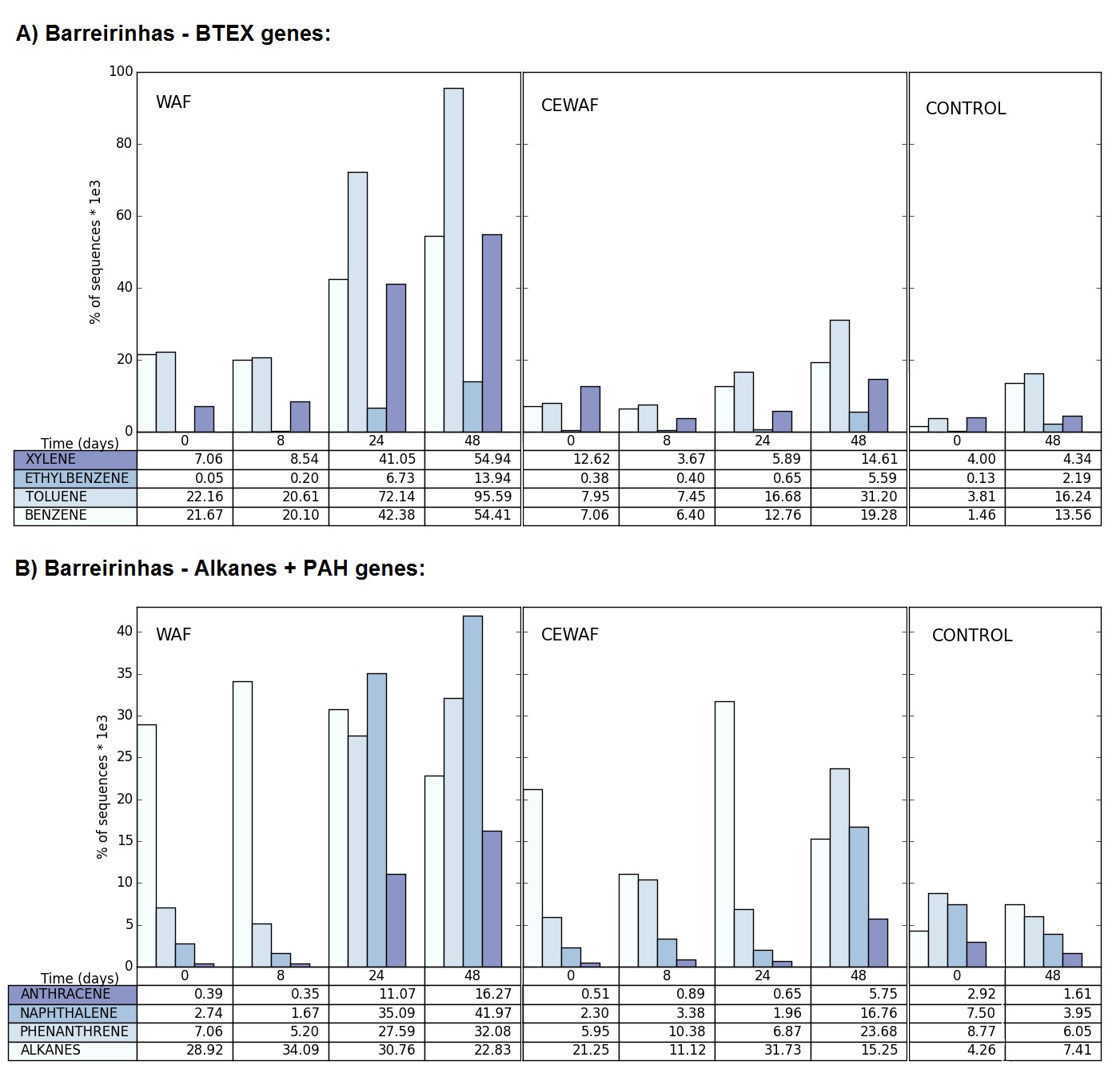


**Supplementary Figure 4.** Proportion (% of sequences X 10^3^) of oil biodegradation related genes determined by metagenomic analyses for BTEX degradation (A) and for alkanes and PAH degradation (B) at Barreirinhas.


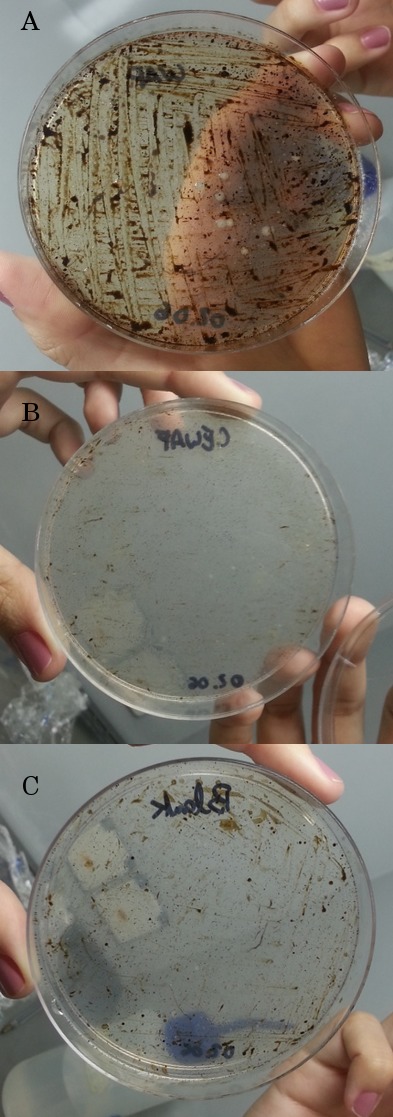


**Supplementary Figure 5.** Colony forming unities (CFU) on mineral medium supplied with oil inoculated with A) WAF (4,0 x 10^3^/mL; n=3), B) CEWAF (2,0 x 10^2^/mL; n=3), and C) Blank (no growth detected) from Barreirinhas experiment in T1 (beginning of the experiment).


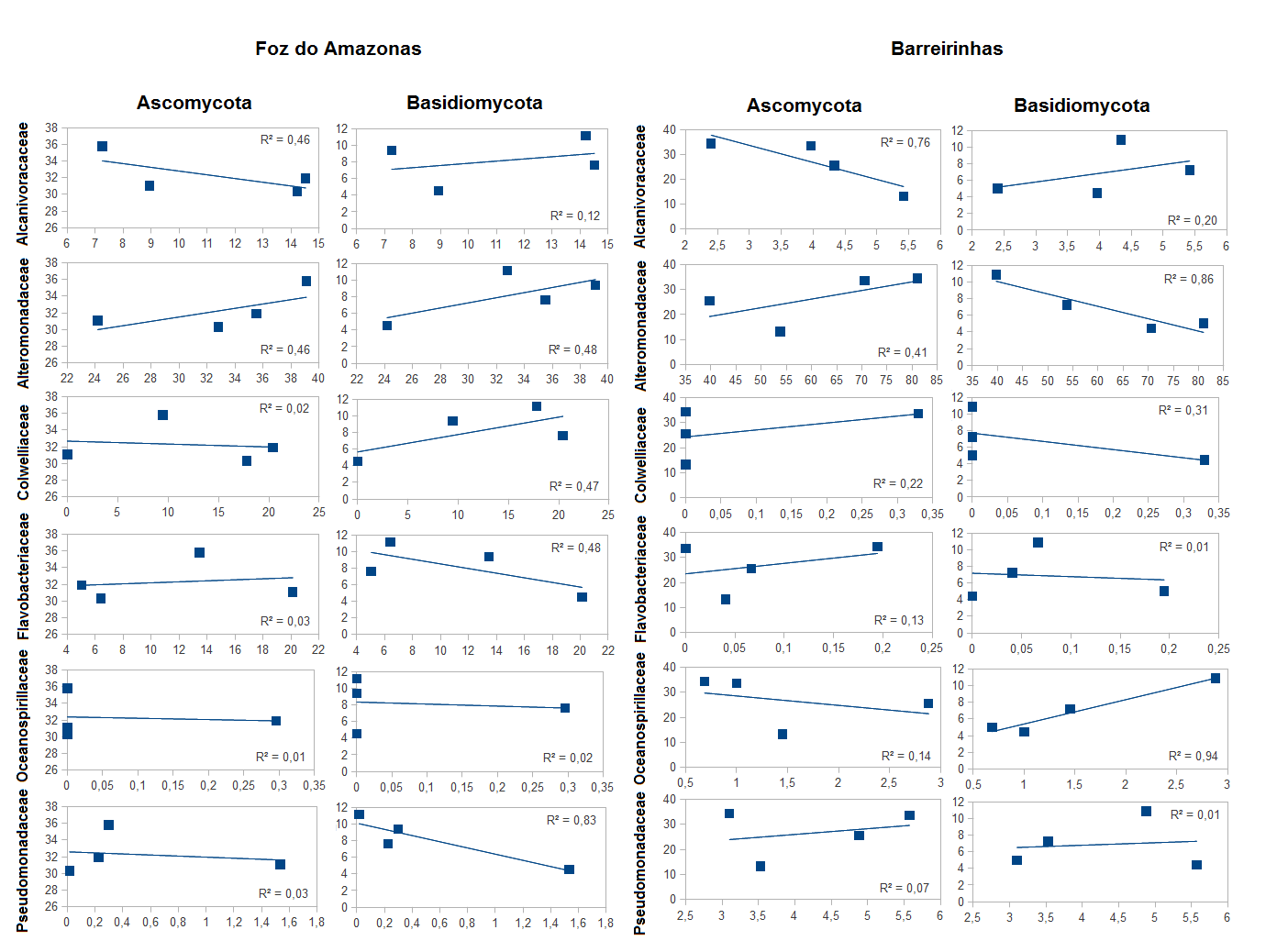


**Supplementary Figure 6.** Linear regression between fungi and known oil degrading bacteria families for WAF samples from Foz do Amazonas and Barreirinhas.


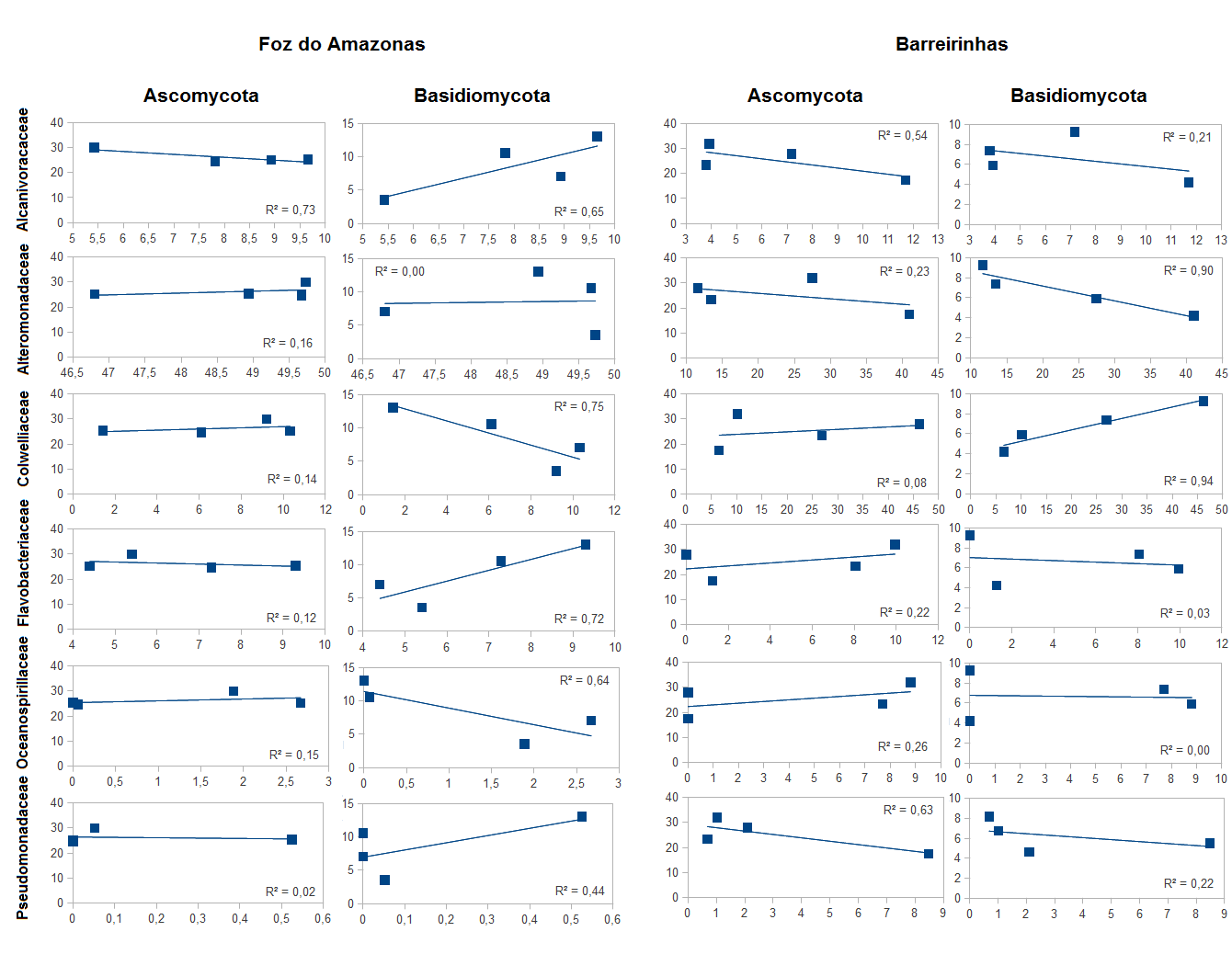


**Supplementary Figure 7.** Linear regression between fungi and known oil degrading bacteria families for CEWAF samples from Foz do Amazonas and Barreirinhas.

**Supplementary Table 1. Metagenomic data information.** For each sample incubated: location, treatment of samples, time of colletion, number of R1 and R2 illumina paired end raw reads, number of sequences after PEAR merge, sample names in MG-Rast and MG-Rast ids.

| Location | Samples | Time | R1 reads (raw) | R2 reads (raw) | sequences (after PEAR) | bp (after PEAR) | MG-RAST ids |
| --- | --- | --- | --- | --- | --- | --- | --- |
| Foz do Amazonas | WAF | T1 | 2137065 | 2137065 | 2032254 | 552675400 | 4681793.3 |
| Foz do Amazonas | CEWAF | T1 | 2105653 | 2105653 | 1870687 | 531993096 | 4681797.3 |
| Foz do Amazonas | WAF | T2 | 1387569 | 1387569 | 1342415 | 335575311 | 4681783.3 |
| Foz do Amazonas | CEWAF | T2 | 1850957 | 1850957 | 1806701 | 442368184 | 4681791.3 |
| Foz do Amazonas | WAF | T3 | 2186280 | 2186280 | 2137983 | 546686691 | 4681785.3 |
| Foz do Amazonas | CEWAF | T3 | 2647494 | 2647494 | 2500714 | 584397239 | 4681789.3 |
| Foz do Amazonas | WAF | T4 | 1964853 | 1964853 | 1683019 | 477542021 | 4681786.3 |
| Foz do Amazonas | CEWAF | T4 | 2499584 | 2499584 | 2323893 | 618451519 | 4681787.3 |
| Barreirinhas | WAF | T1 | 2163627 | 2163627 | 2040086 | 530774543 | 4682147.3 |
| Barreirinhas | CEWAF | T1 | 2517134 | 2517134 | 2352748 | 568404046 | 4682144.3 |
| Barreirinhas | WAF | T2 | 2167652 | 2167652 | 1979919 | 553783683 | 4682149.3 |
| Barreirinhas | CEWAF | T2 | 2205114 | 2205114 | 2014433 | 547400097 | 4682150.3 |
| Barreirinhas | WAF | T3 | 2051202 | 2051202 | 1798016 | 488338233 | 4682151.3 |
| Barreirinhas | CEWAF | T3 | 342435 | 342435 | 305682 | 79659661 | 4682148.3 |
| Barreirinhas | WAF | T4 | 2874785 | 2874785 | 2790102 | 669826661 | 4682152.3 |
| Barreirinhas | CEWAF | T4 | 2100155 | 2100155 | 1861946 | 546592177 | 4682153.3 |

**Supplementary Table 2. Taxonomic annotation.** For each metagenome, percentage of annotated sequences in MG-Rast atributed to each domain of life (Archaea, Bacteria and Eukarya) or to Viruses are shown. Percentage of unclassified sequences are also shown at last collumn.

| Location | Samples | Time | Archaea (%) | Bacteria (%) | Eukarya (%) | Viruses  (%) | unclassified  (%) |
| --- | --- | --- | --- | --- | --- | --- | --- |
| Foz do Amazonas | Control | T0 | 0.0680268474542466 | 99.7301186195892 | 0.0986567368839074 | 0.038198321410827 | 0.0649994746617801 |
| Foz do Amazonas | Control | T4 | 0.0669733753272015 | 99.5266387978417 | 0.188793259667214 | 0.0323842452713834 | 0.185210321892508 |
| Foz do Amazonas | WAF | T1 | 0.0382895199890295 | 99.7411430185855 | 0.0851704317339576 | 0.0476657023380151 | 0.0877313273535044 |
| Foz do Amazonas | CEWAF | T1 | 0.0900601572261718 | 98.1777150116906 | 0.277885823939482 | 1.19765831262648 | 0.256680694517303 |
| Foz do Amazonas | WAF | T2 | 0.0459538606593339 | 99.7812881586472 | 0.0859034005856888 | 0.0444676426561213 | 0.0423869374516236 |
| Foz do Amazonas | CEWAF | T2 | 0.0821585245462213 | 99.7451887139903 | 0.0925645445650066 | 0.0512128733908806 | 0.0288753435076242 |
| Foz do Amazonas | WAF | T3 | 4.67332084605582 | 93.7296761005483 | 0.49897792488817 | 0.229389577399738 | 0.868635551108032 |
| Foz do Amazonas | CEWAF | T3 | 0.0345871162991786 | 99.7745719436491 | 0.0785551721606343 | 0.0529411284862426 | 0.0593446394048406 |
| Foz do Amazonas | WAF | T4 | 0.0530509213389495 | 99.7394468425705 | 0.107610949849467 | 0.0610607978649616 | 0.038830488376102 |
| Foz do Amazonas | CEWAF | T4 | 0.0875306228332091 | 99.5871715668926 | 0.271095824986857 | 0.0126270869052814 | 0.0415748983820149 |
| Barreirinhas | Control | T0 | 2.56574995325597 | 91.1440949361298 | 1.66776730623108 | 3.46025166189944 | 1.16213614248376 |
| Barreirinhas | Control | T4 | 0.192489777232101 | 99.1426401273554 | 0.573238024881471 | 0.0380817613334859 | 0.0535503091975431 |
| Barreirinhas | WAF | T1 | 0.0829407998252833 | 99.4965974914255 | 0.0495361569273685 | 0.315830226982811 | 0.0550953248390571 |
| Barreirinhas | CEWAF | T1 | 0.250617792749428 | 98.9262950309785 | 0.184316789376564 | 0.470418879131149 | 0.168351507764378 |
| Barreirinhas | WAF | T2 | 0.0576118877461199 | 99.727453416783 | 0.0502636930291657 | 0.126195038437692 | 0.0384759640040517 |
| Barreirinhas | CEWAF | T2 | 0.188340917280929 | 99.4034185898552 | 0.137717074100741 | 0.199390058517119 | 0.0711333602460564 |
| Barreirinhas | WAF | T3 | 0.0479601050228422 | 99.7126675850864 | 0.0885182040408835 | 0.0795256843491006 | 0.0713284215008087 |
| Barreirinhas | CEWAF | T3 | 0.063601063524551 | 99.5390118403183 | 0.195107021939976 | 0.170718644197479 | 0.031561430019702 |
| Barreirinhas | WAF | T4 | 0.0552651225768244 | 99.6051000446556 | 0.144271972234385 | 0.0456122058088818 | 0.149750654724298 |
| Barreirinhas | CEWAF | T4 | 0.0879036436351358 | 99.3791441328684 | 0.22681759702874 | 0.231911351212896 | 0.0742232752548332 |

**Supplementary Table 3. Hydrocarbons and oxygen concentrations.** Measures of hydrocarbons for BTEX, PAH and alkanes in μg/L and oxygen in mL/L for WAF, CEWAF and WAF-BLANK treatments in the beginning and in the end (T1 and T4) of both Foz do Amazonas and Barreirinhas experiments. ND, not determined.

|  | Foz do Amazonas | | | Barreirinhas | | |
| --- | --- | --- | --- | --- | --- | --- |
|  | WAF | CEWAF | WAF – BLANK | WAF | CEWAF | WAF – BLANK |
| BTEX (μg/L) | 637.43 – 1.8 | 991.08 – 1.8 | 122.31 – 122.31 | 508.78 – 1.8 | 370.14 – 72.89 | 514.86 – 681.34 |
| PAH (μg/L) | 0.6 – 0.14 | 0.45 – 0.09 | 1.16 – 1.16 | 0.29 – 0.09 | 0.28 – 0.21 | 0.99 – 0.25 |
| Alkanes (μg/L) | ND | ND | ND | 1522.97 – 571.18 | 1331.26 – 429.40 | 2291.14 – 2006.97 |
| O_2_ (mL/L) | 7.1 – 6.2 | 6.6 – 6.4 | ND | 7.5 – 6.8 | 7.8 – 6.7 | ND |
